# Photodynamic Therapy and Immunological View in Gastrointestinal Tumors

**DOI:** 10.3390/cancers16010066

**Published:** 2023-12-22

**Authors:** David Aebisher, Paweł Woźnicki, Klaudia Dynarowicz, Aleksandra Kawczyk-Krupka, Grzegorz Cieślar, Dorota Bartusik-Aebisher

**Affiliations:** 1 Department of Photomedicine and Physical Chemistry, Medical College of the University of Rzeszów, 35-959 Rzeszów, Poland; 2 Students English Division Science Club, Medical College of the University of Rzeszów, 35-959 Rzeszów, Poland; 3 Center for Innovative Research in Medical and Natural Sciences, Medical College of the University of Rzeszów, 35-310 Rzeszów, Poland; kdynarowicz@ur.edu.pl; 4 Department of Internal Medicine, Angiology and Physical Medicine, Center for Laser Diagnostics and Therapy, Medical University of Silesia, Batorego 15 Street, 41-902 Bytom, Poland; akawczyk@sum.edu.pl (A.K.-K.); cieslar1@tlen.pl (G.C.); 5 Department of Biochemistry and General Chemistry, Medical College of the University of Rzeszów, 35-959 Rzeszów, Poland; dbartusikaebisher@ur.edu.pl

**Keywords:** gastrointestinal cancers, photodynamic therapy, anticancer effect

## Abstract

**Simple Summary:**

Many clinical cases of gastrointestinal tumors exist that require the use of high-precision technology for eradication due to their proximity to vital anatomical sites. These sites within the gastrointestinal system are often inaccessible or unsafe for treatment by traditional surgical procedures. Therefore, we reviewed the current literature on the potential of photodynamic therapy (PDT) and associated immunological anti-tumor mechanisms in gastrointestinal tumors. Since its discovery, PDT has emerged as a powerful method for the treatment of skin and esophageal cancers. Traditionally, PDT uses intravenously injected photosensitizers to generate cytotoxic singlet oxygen upon local illumination. Prodrug delivery strategies have shown promise, but the selectivity of the photosensitizer drug in diseased tissue could be improved. Thus, there is a critical need for treatment strategies that enable photodynamic action site-specifically for enhanced tumor destruction.

**Abstract:**

Gastrointestinal cancers are a specific group of oncological diseases in which the location and nature of growth are of key importance for clinical symptoms and prognosis. At the same time, as research shows, they pose a serious threat to a patient’s life, especially at an advanced stage of development. The type of therapy used depends on the anatomical location of the cancer, its type, and the degree of progression. One of the modern forms of therapy used to treat gastrointestinal cancers is PDT, which has been approved for the treatment of esophageal cancer in the United States. Despite the increasingly rapid clinical use of this treatment method, the exact immunological mechanisms it induces in cancer cells has not yet been fully elucidated. This article presents a review of the current understanding of the mode of action of photodynamic therapy on cells of various gastrointestinal cancers with an emphasis on colorectal cancer. The types of cell death induced by PDT include apoptosis, necrosis, and pyroptosis. Anticancer effects are also a result of the destruction of tumor vasculature and activation of the immune system. Many reports exist that concern the mechanism of apoptosis induction, of which the mitochondrial pathway is most often emphasized. Photodynamic therapy may also have a beneficial effect on such aspects of cancer as the ability to develop metastases or contribute to reducing resistance to known pharmacological agents.

## 1. Introduction

### 1.1. Gastric Cancers—Morbidity

Cancers are one of the leading causes of death worldwide. One of the more diverse groups of cancers are those located in the gastrointestinal tract. Currently, there is a significant increase in new cases of this disease [1]. An example of an aggressive cancer of the gastrointestinal tract is esophageal cancer (EC), which is more often diagnosed in men, and is a significant cause of cancer-related mortality worldwide, accounting for 16,910 new cases and 15,910 deaths in the United States in 2016 [2,3]. Nonspecific symptoms can delay a patient’s examination by a physician resulting in an inoperable tumor stage and the presence of metastases in more than 50% of patients, giving EC a poor prognosis [3,4]. More than 95% of new cases of esophageal cancer are adenocarcinoma, which is more common in developed countries, and squamous cell carcinoma which is prevalent in non-industrialized countries. Smoking, obesity, and gastroesophageal reflux disease predispose a person to the development of adenocarcinoma, while achalasia, alcohol consumption, and smoking are risk factors for squamous cell carcinoma [3]. The third most common cause of cancer deaths worldwide is gastric cancer (GC). Common societal risk factors for this disease include high salt intake, a diet low in fruits and vegetables, and H. pylori infection [5]. Gastric cancer should be treated in a multidisciplinary manner. Surgical resection is the primary treatment method with demonstrated treatment efficacy and this is being expanded with adjuvant and neoadjuvant therapies for the treatment of locally advanced lesions. In patients found to have metastases, therapy has unsatisfactory results, with a median survival of about 1 year [6]. Colorectal cancer (CRC), which is the third most common cancer worldwide, is estimated to occur in more patients than cancers of the upper gastrointestinal tract [7]. Its occurrence, mainly in developed Western countries is increasing annually, and it is the fourth most common cause of death among cancer patients [8]. Lifestyles, such as an unhealthy diet, smoking, and alcohol consumption, as well as chronic diseases, age, and environmental factors, predispose a person to the development of this cancer [8,9]. Two pathways lead to the development of colorectal cancer. The first is the multi-step adenoma-carcinoma sequence of mutations of the APC gene, and the second is the development of serrated adenoma to cancer, in which the genetic defect responsible has not yet been determined. Early-stage pre-cancerous adenomatous polyps, as well as advanced cancer, can be asymptomatic, worsening the prognosis, making early diagnosis difficult, and warranting screening in people over 50. Localized colorectal cancer should not be associated with a poor prognosis; however, most cancers are diagnosed at a locally advanced tumor stage or with lymph node metastasis, which is responsible for an unfavorable prognosis. Up to 20% of patients have metastases, most commonly in the liver [10].

### 1.2. Photodynamic Therapy—One of the Treatment Methods

**Figure 1:**
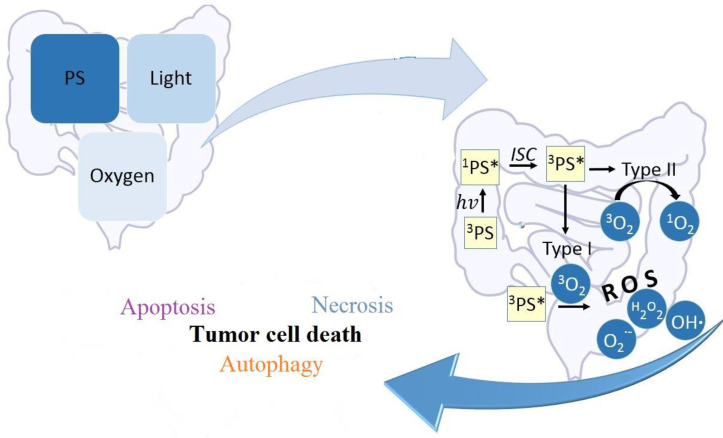
The mechanism of PDT. Most PDT therapies used in clinical settings are based on three components: oxygen, PS, and light. After the application of these three elements, a number of reactions are initiated in the tissue. PS in the ground state becomes excited to singlet state under the influence of light of a specific wavelength. A photosensitizer in the excited singlet state may end up in the excited triplet state as a result of intersystem crossing. A photosensitizer in the excited triplet state can generate reactive oxygen species by electron transfer to an oxygen molecule (Type I). The main components of ROS are superoxide anion, hydroxyl radical, and hydrogen peroxide. In turn, in type (II), energy is transferred from the photosensitizer in the excited triplet state to oxygen in the triplet state, generating singlet oxygen. Both processes have the ability to eliminate cancer cells. Molecular biology describes three main and fundamental mechanisms of cell death: apoptosis, necrosis, and autophagy. PS—photosensitizer, ^3^PS—photosensitizer in the ground state, ^1^PS*—excited singlet state, hv—specific wavelength of light, ^3^PS*—excited triplet state, ISC—intersystem crossing, ROS—reactive oxygen species, O_2_^−^—superoxide anion, OH—hydroxyl radical, ^3^O_2_—triplet state oxygen, ^1^O_2_—singlet oxygen.

The significant increase in new cancer cases worldwide creates the need to discover new and effective therapeutic methods. The search for innovative forms of therapy, including photodynamic therapy (PDT), is ongoing. Photodynamic therapy exploits photodynamic action initiated by the excitation of photosensitizers (PSs) with light and the subsequent interaction of the excited PSs with oxygen to produce reactive oxygen species (ROS) including singlet oxygen (^1^O_2_) [11,12]. Figure 1 presents the mechanism of ^1^O_2_ generation upon excitation of a PS. 

Cell signal transmission depends on the healthy amount of ROS, but when that level is excessively increased, it can result in irreparable cellular damage. ROS produced through PDT oxidizes biological macromolecules in tumor cells, including DNA/RNA and protein, leading to tumor cell death which is known as apoptosis, necrosis, and autophagy. Additionally, ROS in tumor tissue might harm microvascular structures and result in immunogenic cell death. The main signs of apoptosis are shrinking cells and the appearance of vesicles in the cell membrane. Typically, apoptotic cells are surrounded by healthy-looking neighboring cells. 

Apoptosis is characterized by several microscopically detectable changes, which include the most striking condensation of chromatin into well-defined granular masses along the nuclear envelope, cell shrinkage, twisting of cell and nucleus contours, and fragmentation of the nucleus. Eventually, the cell disintegrates into membrane-bound apoptotic bodies that contain, among other things, nuclear debris and that are rapidly removed by neighboring macrophages. During this process, the cell membrane and the membrane surrounding the apoptotic fragments maintain their integrity. In addition, lysosomes remain intact, and therefore, lysosomal enzymes are not released into surrounding tissues. Consequently, there is no accompanying inflammation in apoptosis. 

Necrosis is a non-programmed process, defined as accidental cell death caused by physical or chemical damage. It is characterized by pyknotic nuclei, cytoplasmic swelling, and progressive breakdown of cytoplasmic membranes, leading to cell fragmentation and the release of material into the extracellular space. 

Autophagy is a progressive course of degradation and restoration of cytoplasmic parts. Moreover, it is important for maintaining cell homeostasis and development. It is a physiological cycle in which the cytosol and whole organelles become surrounded by a double layer of vacuoles, known as the autophagosome. As a result of the fusion of the lysosome with the autophagosome, the autophagosome becomes damaged. Studies have confirmed that PDT can induce pathways of apoptosis, autophagy, mitotic catastrophe, and necrosis. The phototoxic effect of PDT leads to photodamage due to irreversible degradation of cell membranes and organelles. Induction of multiple cell death pathways is considered to be a useful feature of PDT because it enhances the photo-killing of cancer cells resistant to a particular cell death pathway. At the level of molecular biology, PDT induces concentration-dependent cell death mechanisms, physicochemical properties, subcellular localization of PSs, oxygen concentration, and the appropriate wavelength and intensity of light. Cell-type-specific properties can affect the mode and extent of cell death. Where the PS enters the cell depends on the chemical properties of each compound. Hydrophobic molecules can diffuse rapidly into plasma membranes, whereas more polar molecules tend to be internalized by endocytosis or assisted transport by lipids and serum proteins. Most PSs are found in organelle membranes, but in general, the cellular localization of PSs includes the endoplasmic reticulum (ER), mitochondria, Golgi complex, lysosomes, and cell membrane. Photogenerated ROS are very short-lived and have limited diffusion distance in biological systems. Therefore, the subcellular location of a PS is often the site where the generated ROS will cause more damage through the activation of cell death mechanisms. Mitochondria, the main intracellular target of PDT, plays a critical role in apoptosis by controlling the release of crucial factors involved in this process.

### 1.3. Overall Cellular Response to PDT

**Figure 2:**
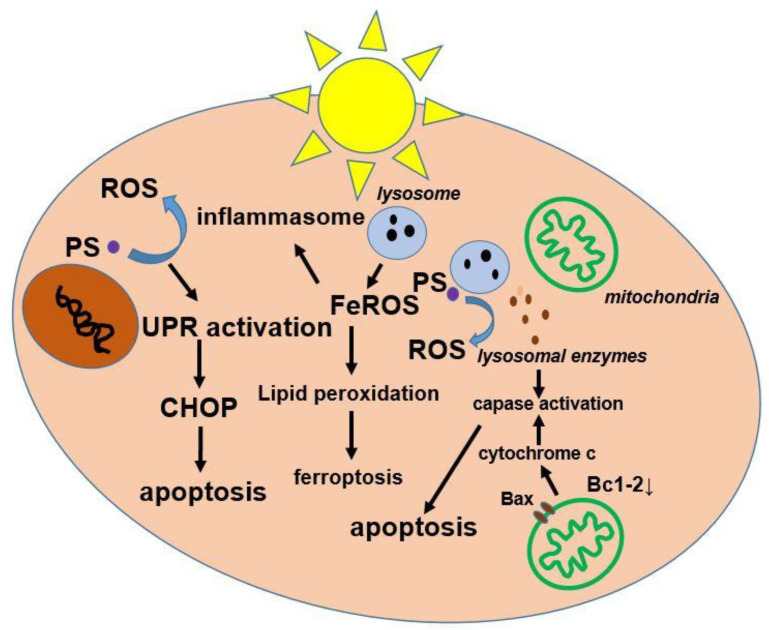
Diagram of activation of processes in cells as a result of PDT activity. Initially, PS penetrates the cancer cells and accumulates in the mitochondria. Upon activation of PS with laser light of a specific wavelength, ROS photogeneration and destruction of the apoptotic protein Bcl-2 occur, which causes the permeabilization of the outer membrane of the mitochondria. As a consequence, cytochrome c is released from the mitochondria into the cytosol, enhancing the apoptotic signal by activating caspases. Most often, PS is found in the plasma membrane, endoplasmic reticulum, mitochondrion, or lysosome. Depending on its location, when activated with light, it can directly damage the plasma membrane causing unregulated necrosis or lead to one or more mechanisms of regulated cell death. UPR: unfolded protein response; Fe: iron; ROS: reactive oxygen species; CHOP: pro-apoptotic transcription factor.

Numerous studies have demonstrated the effectiveness of PDT as a cancer therapy and also in the treatment of many non-oncological diseases, e.g., dermatoses [13,14,15,16]. In the presence of oxygen, the reactions of photo-excited PSs in tumor tissue result in direct cell death through various pathways, the induction of an inflammatory response, and, especially in the case of vascular photosensitizers, the destruction of blood vessels supplying the tumor [17,18,19,20,21]. The phenomenon of photon absorption is key to the excitation of photosensitizers (PSs) to a higher energy level and the formation of a triplet state, which is responsible for energy transfer or electron transfer leading to the production of ROS including ^1^O_2_ [19]. Generating ^1^O_2_ in concentrations sufficient to destroy a hypoxic tumor is a major technological challenge. Most of the clinical experience with gastrointestinal PDT involves patients who are considered to be at risk of poor surgical outcomes, and follow-up reports are limited [22]. Despite the demonstrated efficacy of PDT and knowledge of its underlying mechanism of action, elucidation of the exact mechanisms by which it leads to cell death is ongoing. The best documented cytotoxic effects of PDT on organelles are associated with photodamage to mitochondria and lysosomes (Figure 2).

A type of non-apoptotic cell death is ferroptosis [23]. Its basic characteristic is the gradual loss of mitochondria in response to the administered therapy [24]. In turn, inflammasomes are multi-proteins that contribute to the activation of the inflammatory process and, consequently, to cell death, called pyroptosis [25]. PSs should not accumulate in cell nuclei to prevent the formation of resistant cells [26]. It has been shown that at the cellular level, PDT-induced cell death subroutines may or may not be random [27]. Accidental cell death is an uncontrolled form of death characterized by the gradual loss of cell membrane integrity and swelling of cell organelles [28]. Regulated cell death (RCD) is triggered by the activation of one or more signal transduction modules, such that it can be modulated in some sense pharmacologically or genetically. There are also PDT-associated RCD subroutines that involve apoptosis and various mechanisms of regulated necrosis, including necroptosis and autophagy-dependent cell death [29]. 

### 1.4. Merits and Defects of PDT

#### 1.4.1. Merits

For the treatment of gastrointestinal cancers, PDT has found application mainly in the treatment of lesions located in the esophagus. In addition, it has been shown in studies that PDT is indicated not only in the treatment of already-formed cancer but also in Barrett’s esophagus [30]. The origins of PDT used to treat esophageal cancer include the palliative treatment of patients with obstructive esophageal cancer [31]. Moreover, PDT is being used to treat superficial esophageal cancers characterized by difficulty in endoscopic treatment, and this indication has already been approved for treatment in Japan [32]. In patients in whom local radiotherapy has not achieved the intended therapeutic goals, and in whom treatment by other means may be insufficiently effective, PDT using second-generation photosensitizers is indicated [33]. An example of the strength of PDT is that the cure of early mucosal disease is possible after a single endoscopic procedure [34]. Photodynamic therapy using the photosensitizer sodium porfimer (Photofrin^®^) was approved in the United States in 1995 for use in patients with advanced esophageal cancer [32]. Photodynamic therapy has a favorable side-effect profile, is less invasive, and minimizes systemic toxicity, making it well tolerated by patients [35,36]. Moreover, PDT does not impair fertility and does not affect pregnancy [37]. Enhancing the systemic immune response against cancer may increase the effectiveness of PDT as well as act synergistically with other forms of therapy [38]. 

#### 1.4.2. Defects

The use of PDT is limited to the treatment of flat superficial lesions that are usually accessible endoscopically due to the limited tissue penetration depth of light [39]. At least partially, this problem can be solved by implantable devices or lasers in the near-infrared range, enabling tissue penetration up to 3 cm [40,41]. Another aspect is that the usefulness of PDT is also limited by hypoxia which is typical of many tumors that limit photodynamic action [42,43]. Additionally, reducing tumor oxygenation may promote proliferation and metastasis [43]. Standard guidelines for PDT treatment protocols are still not available, which makes the selection of parameters difficult and affects the quality of treatment [44].

Recent research has focused on improving the effectiveness of PDT. Zaigang Zhou et al. synthesized nanoparticles capable of dually disrupting the PD-1/PD-L1 axis and reversing tumor hypoxia [45]. In turn, Liao W. et al. described the synthesis of a nanogel with the ability to increase the production of ROS in cancer cells [46]. Recent reports have also demonstrated the potential of using PDT based on synthetic hypericin in the treatment of early stages of early-stage cutaneous t-cell lymphoma (Mycosis Fungoides) [47]. It was also found that 5-ALA PDT achieves high effectiveness in the treatment of low-grade squamous intraepithelial lesions with high-risk HPV infection and that the effectiveness of 5-ALA PDT in the treatment of actinic keratosis is increased by microneedling and cryotherapy [48,49]. The aim of this study is to review studies on the treatment of gastroenterological diseases with PDT and its immunological effects.

## 2. Materials and Methods

The literature search, which focused on the immunological mechanisms induced by PDT in the treatment of gastrointestinal cancers, was conducted using articles from PubMed, ScienceDirect, Web of Science, and Google Scholar from 1990 to September 2023. The authors of this review worked according to an agreed framework, selecting articles based on their title, language, abstract, and access. Duplicate works have been removed. The review included papers describing the immunological view of photodynamic therapy in the treatment of gastrointestinal cancers, such as esophageal, stomach, and colon cancer.

## 3. Literature Review

### 3.1. Esophageal Cancer

The anti-tumor effect of PDT in esophageal cancer is due to a combination of direct cell damage, destruction of tumor blood vessels, and activation of the immune response [50]. However, the exact mechanisms of action of PDT have not yet been precisely researched and established. The mechanism of photosensitizer accumulation in cancer cells is also insufficiently understood. 

One study suggests that in the case of Photofrin-II, the mechanism responsible for the accumulation of the photosensitizer in cancer cells is the direct uptake of this compound by the cells, while others negate these conclusions [51,52]. It has been shown that after administration of 5-alpha-aminolevulinic acid, porphyrins accumulate in greater amounts in Barrett’s epithelium and esophageal adenocarcinoma, which results from an imbalance between the activity of porphobilinogen deaminase and ferrochelatase enzymes [53]. 

Photodynamic therapy causes cell death by apoptosis and necrosis and induces autophagy and pyroptosis of esophageal cancer cells [54,55,56,57,58]. In a study by Shi Y. et al., PDT using sinoporphyrin sodium (DVDMs-PDT) was shown to induce apoptosis and autophagy of Eca-109 esophageal cancer cells [54]. By inducing the formation of reactive oxygen species in Eca-109, PDT led to the activation of p38MAPK and JNK kinases and HO-1 heme oxygenase proteins responsible for cellular responses to stress [54,59,60,61]. In Eca-109 cells, apoptosis is also induced by ALA-PDT, stopping the cell cycle in the G0/G1 phase and increasing the level of the pro-apoptotic Bax protein while decreasing the anti-apoptotic Bcl-2 [62,63]. Despite the observed increased levels of apoptosis and caspase-3 activity in esophageal adenocarcinoma cells, PDT using Photofrin-II has not been shown to be responsible for these differences [55]. 

Necrosis of Eca-109 esophageal cancer cells was induced by PDT with hematoporphyrin, while significantly increasing the level of malondialdehyde (a product of peroxidation of omega-6 fatty acids) without an increase in the expression of caspase-3, a key proenzyme in the apoptosis process [64,65]. 

By inhibiting the last enzyme involved in glycolysis, pyruvate kinase (PMK-2), and consequently activating caspase-8 and caspase-9, ultimately leading to the release of gasdermin E (GSDME), PDT can induce pyroptosis of esophageal squamous cell carcinoma cells [66]. A reduction in PKM-2 activity was also observed when examining the effect of ALA-PDT on the Warburg effect. It was shown that in esophageal cancer cells, glucose uptake was inhibited within 4 h after ALA-PDT; however, after 24 h, a significant increase in the expression of this enzyme and glucose uptake was observed [67]. ALA-PDT enhances the effect of the EGFR inhibitor AG1478 and the PI34 inhibitor LY294002, significantly reducing the expression of EGFR/PI3K and PI3K/AKT proteins, leading to a synergistic reduction in the growth and migration ability of Eca-109 esophageal cancer cells in vitro [68]. 

Photodynamic therapy increased NF-κB activity and HIF-1α and VEGF gene expression in vitro and *in vivo*, which may maintain their proliferation, protect against apoptosis, and promote tumor development. Dihydroartemisinin (DHA) may enhance the effect of PDT on esophageal cancer cells [69,70,71]. Zhou et al. examined the mechanism of action of DHA and showed that it was at least partially due to the deactivation of NF-kB [72].

### 3.2. Stomach Cancer

There are a very limited number of reports on the mechanism of action of PDT on gastric cancer cells. 

One study showed that the mechanism underlying gastric-cancer-specific porphyrin accumulation is closely related to both nitric oxide (NO) and heme carrier protein-1 (HCP-1). Moreover, NO has been found to inactivate ferrochelatase, and thus, intracellular porphyrin levels in cells are increased following administration of a NO donor after 5-aminolevulinic acid treatment, and HCP-1 transports not only heme but also other porphyrins. Since NO stabilizes hypoxia-inducible factor (HIF)-1α, causing up-regulation of heme biosynthesis, HCP-1 expression may be increased by stabilizing HIF-1α, which affects the efficiency of porphyrin accumulation by cancer cells [73]. 

One study tested the dependence of the type of gastric cancer cell death induced by PDT using the chlorin-based photosensitizer DH-II-24 on dose level. It was shown that through intracellular free radical production and an increase in intracellular Ca^2+^ ion levels, low-dose PDT (LDP) led to apoptosis, while high-dose PDT (HDP) induced a massive and prolonged increase in intracellular Ca^2+^ ion levels and was thus responsible for inducing necrosis. Moreover, LDP activated caspase-3 [74]. 

It was observed that 5-ALA-PDT applied to human gastric cancer xenografts in vivo caused the apoptosis and necrosis of cells, and in histological examination, most of the tumor blood vessels were hyperemic [75]. It has been shown that PDT via the photosensitizer Photofrin in the MKN45 gastric cancer cell line within 15 min leads to an increase in the activity of caspase-3 and caspase-9 and chromatin condensation. The reduction in rhodamine 123 uptake begins after 30 min and induces mitochondrial damage and apoptosis after 60 min [76]. Moreover, due to its ability to activate the immune system, PDT has a specific effect on metastatic lesions [77]. The effect of PDT on gastric adenocarcinoma cells was studied in patients receiving immune checkpoint inhibitors. Immune cell infiltration increased in tumors after PDT, which is associated with the up-regulation of the B2M gene, which is lost in tumor cells. TCR analysis revealed specific clonal expansion after PDT in cytotoxic T cells but constriction in Treg cells [78,79].

### 3.3. Colon Cancer

The largest number of reports on the effects of PDT on gastrointestinal cancer concern colorectal cancer treatment. However, the exact sequence of reactions occurring after PDT has not yet been fully explained [80]. It has been established that PDT leads to the direct killing of cancer cells by ^1^O_2_ and the indirect killing of cells through damage to blood vessels and the induced immune response [81]. The effectiveness of PDT itself depends on the concentration of the photosensitizer in the cell, but it has been shown that precise intracellular localization has an additional impact on the way in which the therapy causes damage. Moreover, the degree of differentiation of cancer cells also affects the effectiveness of therapy. It was shown that well-differentiated tumor cells had a better response to PDT using protoporphyrin IX (PpIX) than less differentiated cells [82]. Research results indicate that the internalization of a photosensitizer may be the result of partitioning, pinocytosis, and endocytosis, and the target place of its accumulation in the cell is different for different photosensitizers [83,84]. In the case of PpIX, it was found that the tumor-preferential accumulation of this compound is influenced by the difference in activity between porphobilinogen deaminase and ferrochelatase [85]. Photodynamic therapy causes the death of colorectal cancer cells by apoptosis and necrosis [86,87,88,89,90,91,92,93,94,95,96,97,98,99,100,101,102,103,104,105]. 

One of the most important mechanisms of apoptosis triggered by PDT appears to be the mitochondrial pathway. PDT, using hexaminolevulinaine as a photosensitizer, leads to the loss of mitochondrial membrane potential, the release of cytochrome c from mitochondria into the cytosol, and the rapid activation of caspase-9 and caspase-3 and consequently to the apoptosis of 320 DM colon cancer cells [97]. Identical observations were made in the case of PDT with silicon (IV) phthalocyanine [91]. It has been shown that the calcium signal plays an important role in the apoptosis of SW480 cells induced by PDT with the pre-photosensitizer 5-ALA [78]. However, the role of this signal may also contribute to the failure of PDT, as it induces the activation of the ERK pathway, which plays a key role in the survival and development of cancer cells. Calcium ions released from the endoplasmic reticulum were found to result in an increase in the expression level of the chaperone protein GRP78, which in many cancer models, both in vitro and *in vivo*, confers a growth advantage and drug resistance to solid tumors [87,88,91,92]. 

Another study highlighting the involvement of the mitochondrial apoptosis pathway is the study by Guoqing Ouyang et al. who showed that PDT with PpIX led to an increase in the expression of the pro-apoptotic protein bax and caspase-3 while decreasing the expression of the anti-apoptotic bcl-2 [96]. It was shown that cell lines with cytosolic or mitochondrial localization of PpIX were characterized by a loss of mitochondrial transmembrane potential, which led to growth arrest [82]. In turn, in the case of PDT with pyropheophorbide methylester (PPME) that accumulates in the endoplasmic reticulum/Golgi apparatus and lysosomes, it was not demonstrated that transmitters such as calcium ions, Bid proteins, Bap31, phosphorylated Bcl-2, and caspase-12 were involved in triggering the release of cytochrome c from mitochondria when provoking apoptosis [75]. The loss of mitochondrial functionality and therefore apoptosis was also induced by PDT using [Ir-b]Cl and [Rh-b]Cl complexes and PpIX attached to triphenylphosphonium (TPP), which has the ability to target mitochondria [90,100]. Moreover, it is assumed that the leakage of lysosomal protease into the cytosol may also be involved in the induction of apoptosis [99]. 

The effect of PDT on gene expression, which can contribute to resistance, also appears to be important. A study by H. Abrahamse et al. tested the effect of photodynamic therapy on the expression of pro-apoptotic and anti-apoptotic genes in DLD-1 and Caco-2 colon cancer cells. In the case of DLD-1 cells, with increased tumorigenicity, apoptosis was observed with the up-regulation of 3 genes and down-regulation of 20 genes, and these cells were found to have an increased risk of resistance. Caco-2 cells responded better to PDT, and the up-regulation of 16 genes and down-regulation of 22 were observed in these cells [94]. As mentioned earlier, PDT can also cause necrosis of colorectal cancer cells; however, there are no precise reports on the specific mechanism by which this cell death occurs. One study on HT29 colon adenocarcinoma cells showed that the predominant type of cell death provoked by PDT with Foscan^®^ was not apoptosis but necrosis and that changes in mitochondrial membrane potential and cytochrome c release were responsible for cell photoinactivation. HT29 multicellular spheroids loaded with Foscan^®^ showed significantly higher anti-tumor activity at equivalent light doses and the lowest fluence applied. At the lowest fluence rate, and at fluences of moderate levels, 65% cell death was observed via apoptosis. It was also found that the level of caspase-3 activation was not affected by the use of higher fluence values (at identical levels of photocytotoxicity) [106]. It is known that membrane-bound PpIX induces loss of membrane integrity and subsequent necrosis and that 21-selenaporphyrin probably induces necrosis through the endothelial cells of newly formed tumor vessels [82,104]. Necrosis may also be induced by other photosensitizers, e.g., Soranjidol and Rubiadin [103].

So far, it has been established that cellular interactions in the tumor microenvironment also participate in the induction of cancer cell death. It has been shown that due to their plasticity, macrophages residing in or recruited from the tumor can enhance tumor development by promoting tumor cell migration and endothelial stimulation. The increased cytotoxicity of PDT mediated by the production of nitric oxide, interleukin-6 (IL-6), and tumor necrosis factor alpha was in turn achieved in the presence of non-resident macrophages with a strong anti-tumor phenotype (TNF-α) [107]. In contrast, a study by A. Jalili et al. [102] determined the anti-tumor efficacy of combining PDT with the administration of immature dendritic cells. They found that inactivation of C-26 colon cancer cells after PDT was followed by necrosis and apoptosis. Moreover, there was also an increase in the expression of HSP72/73, HSP90, HSP27, HSP60, HO-1, and GRP78 proteins [101]. It was observed that immature dendritic cells cultured with cancer cells after PDT exhibited the ability to engulf dead cancer cells, acquired functional maturation characteristics, and produced significant amounts of interleukin-12 (IL-12), thereby enhancing the activity of macrophages, NK cells, and monocytes. Moreover, these cells also stimulated the cytotoxic activity of NK cells and T lymphocytes and stimulated their influx into lymph nodes [101,102]. 

It has been shown that PDT can also lead to the systemic induction of anti-tumor immune responses. In order to test the potential mechanism of this phenomenon, the effect of vascular PDT on β-galactosidase antigen-expressing colon adenocarcinomas BALB/c, CT26WT, and CT26. CL25 was studied. It was found that the efficacy of the therapy depended on the level of β-galactosidase expression, as complete cure occurred only in antigen-positive tumors. The destruction of distant metastases was also observed in 70% of the mice tested. It was found that T cells in these mice were able to recognize the epitope derived from the beta-galactosidase antigen and specifically destroy the cancerous antigen-positive cells. In the remaining 30% of mice, the tumor antigen was lost and the metastatic lesions were not cured [108]. The effect of PDT on the ability of cells to migrate and metastasize seems to be significant. It is known that PDT using low concentrations (5 μM) of hyperforin and aristophorin not only inhibits cell cycle progression and induces apoptosis but also reduces the expression of metalloproteinases-2/-9 and cell adhesion potential [89]. Similar observations were found in the case of PDT therapy using m-THPC, which also reduces the colony formation and migration ability of SW480 and SW620 colorectal cancer cells [95]. 

The possible mechanisms of this effect were investigated during PDT involving the photosensitizer chlorin-e6 (Ce6-PDT). It was shown that the therapy led to the inhibition of proliferation, almost complete disappearance of pseudopodia, a decrease in the migration ability of SW480 cells, and an increase in the expression of F-actin, α-tubulin, β-tubulin, vimentin, and E-cadherin. It is assumed that the possible inhibition of cancer cell migration was due to the increased expression of E-cadherin, the loss of which is often observed during metastases, causing the disappearance of pseudopodia and destruction of the cytoskeleton [109,110]. In another study, it was shown that under the influence of Ce6-PDT, the healing and migration rate of SW620 cells was significantly reduced, the pseudopodia of the cells were reduced or disappeared, the original microfilament structure was destroyed, and the expression of F-actin was significantly reduced. The Rac1/PAK1/LIMK1/cofilin signaling pathway, which is one of the main pathways through which Rho GTPases regulate microfilaments, was down-regulated by Ce6-PDT [111]. 

Another aspect of PDT’s action is the ability to reduce the resistance of colorectal cancer cells. As shown by M. Luo et al., PDT with the photosensitizer chlorin-e6 can inhibit oxaliplatin (L-OHP)-induced autophagy while promoting apoptosis and increasing the expression of procaspase-3 protein, while the combination of Ce-6PDT with L-OHP led to the same effects and an increase in the expression of proapoptotic Bcl-2 and reduced the migration capacity of SW620 colorectal cancer cells [105].

**Figure 3:**
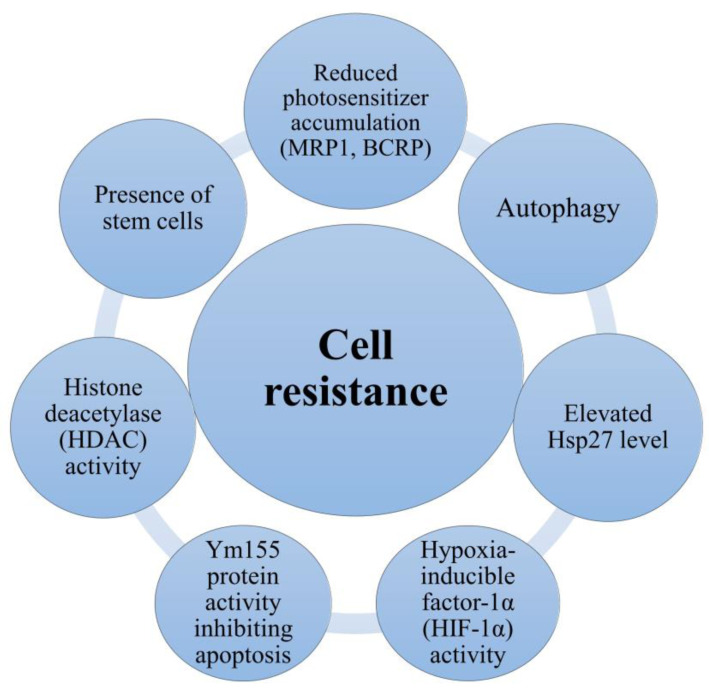
Mechanisms of cell resistance. The basic mechanisms of cellular resistance are presence of stem cells; reduced photosensitizer accumulation (P-gp, MRP1, BCRP); autophagy; elevated Hsp27 level; hypoxia-inducible factor-1α (HIF-1α) activity; Ym155 protein activity inhibiting apoptosis; histone deacetylase (HDAC) activity.

An important aspect of PDT is the possibility of developing tumor resistance to this type of therapy, resulting from cellular responses to stress, hypoxia, or the heterogeneity of PS uptake by individual tumor cells [112,113,114]. It is known that in response to hypoxia, cells can induce HIF-1α-mediated autophagy, leading to increased colon cancer cell survival and reduced cell death after PDT. By binding to hypoxia-responsive elements in the VMP1 promoter, stabilization of HIF-1α has been shown to significantly increase the VMP1-related autophagy process [115]. An important factor involved in tumorigenesis is hypoxia-inducible factor-1 alpha (HIF-1α), which may also contribute to the development of PDT resistance [116,117]. Investigating the effects of PDT using Me-ALA (a pro-drug of PS PpIX) on human colon cancer spheroids, it was discovered that the PDT resistance phenotype was due to the highly regulated transcriptional activity of hypoxia-inducible factor-1α (HIF-1α). Abolishing the RNA interference (RNAi) of HIF-1α reduced the degree of resistance to PDT, while inhibition of the MEK/ERK pathway and removal of ROS abolished the regulation of HIF-1α by PDT [117]. It is known that elevated levels of Hsp27 may play an important role in colorectal cancer cell resistance (Figure 3), as phosphorylation of this protein plays an important role in cytoprotection. 

Studying the effects of Photofrin-PDT on HT29-P14 colon cancer cells, it was found that pathways leading to Hsp27 phosphorylation may contribute to cell resistance to photooxidative damage [118]. 

By examining the effect of YM155, a small molecule inhibitor of survivin expression in HT-29 colorectal adenocarcinoma cells resistant to dynamic phototherapy with hypericin, it was shown that proteins that inhibit apoptosis play a key role in cancer progression and therapeutic resistance [119]. Further, the interaction of hypericin with the mechanisms of elimination of anticancer drugs by cancer cells is unclear. It is known that they are complex. In HT-29 colon cancer cells treated with hypericin, increased activity of multidrug resistance-related protein 1 (MRP1) and breast cancer resistance protein (BCRP) was observed. In contrast, administration of cytochrome P450 enzyme inhibitors led to an increased content of this photosensitizer. Hypericin content in these cells is also known to decrease glycoprotein-p [120]. On the other hand, examining the contribution of the mechanism of export by p-glycoprotein, it was shown that the use of verapamil, a p-glycoprotein antagonist, can reverse the resistance of HRT-18 colorectal cancer cells to PDT with hematoporphyrin, which suggests a significant role of p-glycoprotein in reducing sensitivity to treatment [121]. One study examined the effect of histone deacetylase (HDAC) inhibitors on the development of resistance to PDT with hypericin by colorectal cancer cells. Two chemical classes of histone deacetylase (HDAC) inhibitors have been studied in combination with HY-PDT: the hydroxamic acids Saha and Trichostatin A, and the short-chain fatty acids valproic acid and sodium phenylbutyrate (NaPB). Combining HDAC inhibitors with HY-PDT significantly attenuated the renewed resistance of cancer cells to treatment. The manner, selectivity, and potency of HDAC inhibition depended on the specific inhibitor. To sum up, histone deacetylase may be one of the causes of cell resistance to PDT (Figure 3) [122]. 

Another study showed that a total of 1096 long noncoding lncRNAs were present in HCT116 colon cancer cells treated with PDT. Resistance to PDT was determined by the interaction between Long Noncoding RNA LIFR-AS1, the miR-29a gene, and the TNF Alpha Induced Protein 3 (TNFAIP3) gene. The resistance of HCT116 cells to PDT was due to the role of LIFR-AS1, as it serves as a competitive endogenous RNA for miR-29a, inhibiting its expression and increasing TNFAIP3 expression [123]. Epigenetic changes are known to account for drug resistance in colorectal cancer [124]. At the same time, they are reversible. The regulation of polycomb proteins (PcG), which have the ability to epigenetically silence genes, polycomb group RING finger protein 4 (BMI1) and Enhancer of zeste homolog 2 (EZH2), and the associated cancer progression are potential therapeutic targets. A study of resistance to PDT with hypericin by M. N. Sardoiwala et al. showed that Protein phosphatase 2 mediated the degradation of BMI1 and that inhibition of HMI1 and EZH2 contributed to improved treatment outcomes [125]. 

Stem cells are believed to be resistant to PDT, which may be another reason for the lack of therapeutic efficacy. Through their ability to self-renew cyclically with a long duration of one cycle, they increase resistance to treatment, which contributes to PDT failure and an increased risk of recurrence [126]. A major concern is the ability of cancer cells to acquire resistance to drug treatment. The sensitivity of colorectal cancer cells to treatment may be enhanced by PDT. It was shown that PDT increased the efficacy of L-oxaliplatin (L-OHP) treatment. A multilevel mechanism for this phenomenon has been established, involving the decreased efflux of L-OHP (dependent on multidrug resistance-associated protein 1 (MRP-2)), inhibition of glutathione S-transferase activity and intracellular glutathione, increased DNA double-strand breaks, and decreased expression of DNA excision repair protein (ERCC-1) along with DNA repair endonuclease XPF, involved in the nucleotide excision repair pathway [127]. Photodynamic therapy works synergistically with drugs that block Programmed death-ligand 1 (PD-L1), which may increase the effectiveness of treatment. A study by Z. Yuan et al. showed that the combination can inhibit primary and distant tumor growth, as well as contribute to long-term host immune memory, which prevents cancer recurrence. The mechanism of this interaction has been shown to induce cell death and stimulate a systemic immune response, which can be further promoted by PD-L1 blockade [128]. It is known that the efficacy of PDT of HT-29 colon cancer cells can be enhanced by stimulating apoptosis by administering the specific 5-lipoxygenase inhibitor MK-886. Further analysis of individual ROS groups revealed the effect of increasing MK-886 concentration on peroxide accumulation, which was accompanied by a decrease in the level of hydrogen peroxide in cells. A clonogenicity test revealed impaired colony formation when both agents were combined compared to MK-886 or PDT alone [129]. Figure 3 shows the mechanisms of cell resistance.

Photodynamic therapy does not always lead to complete cure [39]. This phenomenon involves mutations related to the inhibition of apoptosis, drug–drug interactions, increased drug efflux, reduced photosensitizer concentration and light exposure, and local hypoxia [39,130,131,132,133,134]. Much research has been undertaken to develop a new generation of nanomaterial-based photosensitizers that could address this problem [39]. Emerging evidence indicates that overcoming the resistance of cancer cells can be achieved by using photosensitizers with the regulation of ROS production, targeting organelles, nanosubstituted photoactive drugs, and PS delivery nanosystems and combining different types of therapies [131]. Pramual et al. created a new hybrid molecule and demonstrated that it had the potential to deliver a photosensitizer or chemotherapeutic drug for the treatment of multidrug-resistant lung cancer cells [134]. In turn, Qian-Li Ma et al. found that the combination of an ATM inhibitor with PDT has the ability to inhibit the DNA damage response and increase the effectiveness of therapy against PDT-resistant lung cancer cells [110]. Moreover, Deken et al. showed that nanoparticles can induce the regression of tumors overexpressing HER2 during one treatment session, which may be used in the treatment of trastuzumab-resistant cancers [135]. Zhijian Luo et al. created molecules that bind to annexin 1, which improved the cellular uptake of drugs and, consequently, increased cytotoxicity against multidrug-resistant breast cancer cells [136]. As shown by Zhong et al., a properly constructed nanoparticle with palitaxel intended for combined chemo-photodynamic therapy can break the resistance of lung cancer cells to this drug [137]. 

### 3.4. Interaction of PDT with Gastrointestinal Tumor Cells

Modern research methods and advanced drug complexes are being developed to observe and understand the immunological processes occurring in tumor metabolism. One of the main aspects of the analysis is the characterization of the immune response (Table 1), mainly the process of programmed cell death. An example of advanced research assessing the immune response of a tumor is the research conducted by Liu et al. The therapeutic method involving an increase in the infiltration of T lymphocytes has completely revolutionized the therapeutic technique of cancer. Although many metabolic processes are known and investigated, the mechanisms of the tumor’s immune response to PDT remain undiscovered. Additionally, there is still uncertainty about the safety of applied photosensitizers, drugs that target selected cell organelles (i.e., mitochondria). Work by Liu et al. describes an innovatively designed drug that is safe and effective both in vivo and *in vitro*. Drug-assisted PDT has the ability to inhibit tumor growth. Additionally, it alleviates the phenomenon of hypoxia, i.e., tumor hypoxia, by generating a higher number of ROS. The designed multifunctional drug and PDT enable it to influence tumor metabolism and its immune system [138]. Table 1 shows the mechanisms of interaction of PDT with gastrointestinal tumor cells (from the type of accumulation of photosensitizer through the mechanism of destruction to the type of response). 

### 3.5. Clinical Challenges

In the field of PDT in cancer treatment, valuable insights are provided by the dual perspective of photosensitizers undergoing clinical trials and those already in clinical use. Clinical trials are a source of innovation, presenting a diverse range of photosensitizers of different generations. These trials highlight ongoing efforts to improve and expand the potential of PDT. In particular, third-generation photosensitizers demonstrate increased tumor specificity, improved tissue penetration, and reduced side effects, representing significant progress. Challenges such as poor water solubility and aggregation remain, highlighting the complexity of developing effective photosensitizers. 

Certainly, one of the main limitations and challenges of conducting PDT in a clinical setting is the difficult process of monitoring the entire treatment process.

Additionally, uneven and varied distribution of therapy components (such as light and oxygen) may result in numerous side effects. Currently, various types of simulations are practiced to improve PDT in clinical conditions at every stage of treatment (from the application of a photosensitizer to the exposure process and follow-up observations) [139].

Currently, interstitial PDT supported by chemotherapy and immunotherapy is also practiced, introducing a number of combination options in the treatment of gastroenterological diseases. More often, pilot studies are carried out as initial verification and the initial stage of clinical trials.

Despite the high effectiveness of first- and second-generation photosensitizers, new solutions are still being sought. An example of improving treatment results is the use of nanotechnology, i.e., third-generation photosensitizers (Table 2). Currently, ongoing research and the latest literature reports on the use of PDT in gastroenterological diseases give hope for improving the effectiveness, sensitivity, and specificity of treatment. Table 2 shows a review of third-generation photosensitizers in gastroenterological cancers. 

Third-generation photosensitizers (Table 2) and their effectiveness are another challenge in transforming laboratory and preclinical research into clinical trials. The combination of nanotechnology and the process of developing various types of nanoparticles supporting PDT is a promising tool but not free from obstacles and challenges. Many nanocomplexes being developed are in the process of improvement to be safely used in clinical trials. The nanomaterials used are not always free of toxicity, which is why they are subject to control and testing. Due to the fact that some of the research is conducted at an early stage, we can look forward to the future with hope for the development of the PDT technique in the treatment of gastroenterological cancers. We hope that the results of future studies will allow us to improve the effectiveness of clinical trials using PDT as much as possible [144]. 

## 4. Conclusions

Cancer treatment using PDT poses many challenges. One of them is the possibility of cancer cells becoming resistant to this type of therapy. This article presents evidence that mechanisms such as the removal of photosensitizer from cancer cells, induction of autophagy in response to damage, natural increased resistance of tumor stem cells, and, finally, increased presence of various cytoprotective proteins are involved in this process. Interactions between tumor cells and other cells are also an important aspect, as they may contribute to weakening the effect of PDT and even to accelerating tumor development. Further research is necessary to determine the exact mechanisms of action of dynamic phototherapy on gastrointestinal cancer cells, taking into account the type of photosensitizers, the classification of cancers, and their stage of advancement. Understanding the precise impact of PDT on the treatment of this disease may help discover new photosensitizers and their transport mechanism or determine the appropriate, most effective therapeutic regimens. It also seems promising to investigate the mechanisms by which PDT can lead to the activation of the immune system and, as a result, to the treatment of metastases. Moreover, based on this review, it can be concluded that a thorough examination of the mechanisms responsible for cellular resistance to PDT may contribute to the discovery of new therapeutic agents that can inhibit this resistance. In summary, the immunological mechanisms of the action of PDT on gastrointestinal cancer cells are still insufficiently understood, and their detailed examination may contribute to increasing the effectiveness of this therapy. Solutions to certain challenges and application problems emerging in clinical trials are still being sought. The solution turns out to be not only nanotechnology and its possibilities but also designed drugs targeting selected cell organelles. The therapeutic process of cancer is very complex, and the biological and immunological mechanisms initiated as a result of PDT are still not clear and understandable. It is satisfactory that such a difficult and important topic as the immunological aspects of PDT is constantly being explored and addressed.

## Figures and Tables

**Table 1:** Mechanisms of interaction of PDT with gastrointestinal tumor cells.

Mechanism of Interaction of PDT with Gastrointestinal Tumor Cells		
Esophageal cancer	Accumulation of photosensitizer	Imbalance between the activity of porphobilinogen deaminase and ferrochelatase enzymes (5-ALA)
	Mechanism of cell damage	Direct cell damage
		Destruction of tumor blood vessels
		Activation of the immune response
	Type of response and cell death	Apoptosis
		Necrosis
		Pyroptosis
		Autophagy
Gastric cancer	Accumulation of photosensitizer	Dependent on nitric oxide (NO) and heme carrier protein-1 (HCP-1)
	Mechanism of cell damage	Direct cell damage
		Activation of the immune response
	Type of response and cell death	Apoptosis
		Necrosis
Colorectal cancer	Accumulation of photosensitizer	Partitioning
		Pinocytosis
		Endocytosis
		Difference in activity between porphobilinogen deaminase and ferrochelatase (PPIX)
	Mechanism of cell damage	Direct cell damage
		Destruction of tumor blood vessels
		Activation of the immune response
	Type of response and cell death	Apoptosis
		Necrosis

**Table 2:** A review of third-generation photosensitizers in gastroenterological cancers.

Type of Disease	A Type of Third-generation Photosensitizer	Wavelength of Laser Light (nm)	Immunological Effect	References
Colon cancer	porphyrin grafted lipid (PGL) nanoparticles	650	The results confirmed that the designed nanoplatform effectively eliminates differences in oxygen content, which positively affects the process of generating singlet oxygen and the process of weakening COX-2 expression.	[140]
Colon cancer	liposome encapsulating phosphoinositide 3-kinase gamma (PI3Kγ) inhibitor IPI-549 and chlorin e6	660	The proposed therapy significantly limited the development and growth of the tumor by positively affecting the physiology of dendritic cells and T lymphocytes.	[141]
Colorectal cancer	CD133-Pyro	670	The study showed that the designed composite increases ROS production and induces cell death.	[142]
Colorectal cancer	Sinoporphyrin sodium (DVDMS)	635	The therapy induced programmed cell death, among others, by generating the caspase pathway in CX-1 cells.	[143]

## Data Availability

The data can be shared up on request.
